# Effect of the parental origin of the X-chromosome on the clinical features, associated complications, the two-year-response to growth hormone (rhGH) and the biochemical profile in patients with turner syndrome

**DOI:** 10.1186/1687-9856-2013-10

**Published:** 2013-06-04

**Authors:** Francisco Álvarez-Nava, Roberto Lanes, José Miguel Quintero, Mirta Miras, Hugo Fideleff, Verónica Mericq, Henry Marcano, William Zabala, Marisol Soto, Tatiana Pardo, Lisbeth Borjas, Joalice Villalobos, Peter Gunczler, Nancy Unanue, Natalia Tkalenko, Adriana Boyanofsky, Liliana Silvano, Liliana Franchioni, Miriam Llano, Gabriel Fideleff, Miriam Azaretzky, Martha Suarez

**Affiliations:** 1 Unidad de Genética Médica, Universidad del Zulia, Maracaibo, Venezuela; 2 Unidad de Endocrinología Pediátrica, Hospital de Clínicas Caracas, Caracas, Venezuela; 3 Departamento de Endocrinología y Laboratorio, Hospital de Niños de la Santísima Trinidad, Córdoba, Argentina; 4 Unidad de Endocrinología, Hospital T Álvarez, Buenos Aires, Argentina; 5 Instituto de Investigaciones Materno Infantil, Facultad de Medicina, Universidad de Chile, Santiago, Chile; 6 División de Endocrinología, Hospital Domingo Luciani, Caracas, Venezuela; 7 Departamento de Pediatría, Hospital de Especialidades Pediátricas, Maracaibo, Venezuela

**Keywords:** Parental origin of the X-chromosome, Turner syndrome, Genomic imprinting, rhGH

## Abstract

**Background:**

It is possible that genes on the X chromosome are expressed differently depending of its parental origin. The objective of this study was to determine the influence of the parental origin of the X-chromosome on phenotypic variability, response to rhGH and on the biochemical profile of TS patients.

**Methods:**

This was a cross-sectional multicenter correlational study carried out over three years in six Latin-American university hospitals. Unrelated 45,X TS patients (n =  93; 18.3 ± 8.5 years )) were evaluated. A subgroup (n =  34) of the patients were prospectively treated with rhGH over two years. DNA profiles of patients and their mothers were compared to determine the parental origin of the retained X-chromosome through 10 polymorphic X-chromosome-STRs. The association with clinical features, biochemical profiles and anthropometric data at the beginning and after two years of rhGH treatment was determined.

**Results:**

Seventy two percent of patients retained the maternal X chromosome (Xm). A trend towards significance between maternal height and patients final height (p ≤ 0.07) in 45,Xm subjects was observed. There was no correlation between paternal height and patient height. No differences were detected between both groups in regard to dysmorphic features, classical malformations or increase in the height-SDS after rhGH. There were higher levels of triglycerides, total and LDL cholesterol in patients >20 years who retained the Xm.

**Conclusions:**

The parental origin of the retained X chromosome may influence lipid metabolism in TS patients, but its effect on growth seems to be minimal. No parental-origin-effect on the phenotypic features, associated anomalies and on the growth response to rhGH was found in 45,X TS individuals.

## Introduction

Turner syndrome (TS) is defined as the combination of short stature, gonadal dysgenesis, typical visible somatic dysmorphic stigmata, and urinary, cardiovascular, metabolic and skeletal abnormalities associated with a missing or structurally abnormal second sexual chromosome [1]. This disorder is one of the most common human chromosomal anomalies, occurring in approximately 1:2500 live female births. More than 50% of TS patients have an apparent non-mosaic 45,X karyotype and a wide and variable spectrum of clinical features and associated complications of TS are present even in the individuals with non-mosaic pure 45,X karyotypes.

The absence of the second X-chromosome in TS leads to a loss of expression of either paternally or maternally derived genes on the X-chromosome, depending of the X chromosome retained [2]. The haploinsufficiency of genes that are normally expressed from two sexual chromosomes is the most plausible mechanism to explain the TS phenotype. However, the absence of the second sexual chromosome has led to the speculation that there may be genes present on the X chromosome which are expressed differently depending upon whether they are maternally or paternally derived [3]. Thus, both the haploinsufficiency of genes on the two sexual chromosomes and the parental origin (monoallelic expression of the imprinted genes) may play a part in the broad and variable clinical features spectrum seen in TS. Therefore, TS could be a model for understanding the role of genomic imprinting on the clinical features and putative imprinted genes on the X-chromosome.

A continuing controversy remains regarding the impact of the retained X-chromosome on the clinical features of TS according to their parental origin, in particular with regards to height, deafness, cardiovascular and metabolic abnormalities and to the growth response to rhGH. In the context of this controversy, the current study was performed to evaluate the effect of the parental origin of the X-chromosome on the clinical features, auxological data, associated complications, lipid metabolism and response to rhGH in a group of patients with TS and a non-mosaic 45,X karyotype.

## Subjects and methods

This is a cross-sectional multicenter correlational study carried out in 6 Latin-American university hospitals over the past three years. The study protocol was approved by the medical ethics institutional review board of all participating hospitals (Unidad de Genetica Medica, Universidad del Zulia; Unidad de Endocrinologia Pediatrica, Hospital de Clínicas Caracas; Departamento de Endocrinología y Laboratorio, Hospital de Niños de la Santísima Trinidad; Unidad de Endocrinología, Hospital T Álvarez; Instituto de Investigaciones Materno Infantil, Facultad de Medicina, Universidad de Chile; División de Endocrinología, Hospital Domingo Luciani; and Departamento de Pediatría, Hospital de Especialidades Pediátricas). Initially, unrelated prepubertal patients with TS (n =  100) examined at our hospitals were enrolled in this study along with their mothers. However, the study group was reduced to 93 patients after a hidden mosaicism in the second X-chromosome was detected by molecular analysis in duplicate blood samples in seven subjects. Patients included in this study were from the following countries: Venezuela (n =  55) Argentina (n =  29), and Chile (n =  9). The diagnosis of TS was based on typical clinical features and on cytogenetic analysis. Only patients with a non-mosaic 45,X karyotype were included and the average number of analyzed metaphases was 50. A subgroup (n =  34) of the patients that fitted eligibility criteria for rhGH treatment was analyzed both longitudinally and prospectively. We have used the following eligibility criteria for rhGh treatment: 1) age over 2 years old; 2) girls with a height below the third percentile in normal height curves, decreased growth velocity and without significant spontaneous catch-up growth; and 3) no previous administration of growth-stimulating therapy or of estrogen replacement. Compliance was determined by the clinical response to rhGH therapy (significant increase in their linear growth and in their growth velocity following rhGH administration). In some of the girls, compliance was also determined by significant increases in serial IGF-1 measurements during rhGH therapy. With respect to parental age, chronological age, height-SDS, weight-SDS, BMI-SDS, bone age delay, and parental origin of retained X-chromosome, no statistical difference was found between patients of different countries at the beginning of the study. Thus, these country subgroups were pooled as one group. Written informed consents for DNA sampling and for participation in the study were obtained from parents or adult patients before beginning the study.

Each patient was examined by two clinicians in each center. Clinical data were collected from the patients using a previously designed standardized checklist for TS which included more than 100 variables and recorded. The standardized checklist will be sent upon request. Clinical features encompassed facial dysmorphic stigmata and ophthalmologic, auditive, thoracic, skeletal and cutaneous signs. In addition, cardiovascular, renal metabolic and endocrinological abnormalities were prospectively evaluated. Serum fasting levels of glucose, total-, LDL-, VLDL- and HDL-cholesterol and triglyceride concentrations were measured in all subjects by the enzymatic method. Measurements were performed directly in each of the 6 reference hospitals. When a patient was receiving HRT, this was discontinued 3 months prior to assessment. Imaging studies performed included renal and cardiac ultrasonography. All clinicians were blinded to the results of the molecular analysis.

The mean age for the whole group was 18.3 ± 8.5 years (range 0.4-46 years ). Auxological evaluation was performed by trained physicians using standard techniques and included measurements of weight (expressed in kg and in standard deviation scores (SDS)), parental height, standing height expressed in cm and transformed into SDS according to national growth chart references for each country (patient value minus mean age- and sex-matched control values (cm) divided by the corresponding SD according to age) in order to evaluate all patients from different countries. The mid-parental height was calculated as paternal height minus 12.5 cm plus maternal height divided by 2, then converted to SDS using national normative height data for females and males at 19 years of age. To analyze the growth response to rhGH, anthropometric data were evaluated at the start of rhGH therapy and every 3 months thereafter for 2 years . Growth velocities, total height gain and differences in the increment were calculated [4]. Recombinant human growth hormone (rhGH) was administered at a mean dose of 37 ± 5 μg/kg/day seven days a week by subcutaneous injection for a two year period. Body mass index (BMI) was expressed as SDS. Left hand and wrist radiographs were assessed prior to starting therapy and then yearly for bone age determination using the methods of Greulich and Pyle.

### Molecular analysis

Isolation, quantification and purification of genomic DNA, selection of Short Tandem Repeats on the X-chromosome (X-STRs), PCR amplification, and fragments analysis are described in Additional file 1. Briefly, DNA samples were obtained from a few drops of venous blood placed on the filter paper from patients and their mothers. Genomic DNA was extracted and quantitated using the DNA IQ™ System kit (Promega, Madison, WI, USA). PCR conditions were optimized in our laboratory using the QIAGEN Multiplex PCR kit according Gusmao et al. and Edelman et al. [5,6]. The X-STRs were selected due to 1) their location on both Xp and Xq; 2) their high degree of heterozygosity (between 66 to 82%); and 3) their high discrimination power obtained in Latin-American populations, both in males (≥1 in 5 × 105) and females (≥1 in 3 × 109), as well as high mean exclusion chance in father/daughter duos (≥99.953%) and in father/mother/daughter trios (≥99.999%) [5,6]. Separation and detection of the different amplicons generated during the PCR was performed in an ABI PRISM 310 Genetic Analyzer (Applied Biosystems). The parental origin of the single X chromosome was assigned as maternal when the patient and her mother shared an allele at each of the ten loci tested using the X-STRs decaplex. Paternal DNA was excluded to avoid potential ethical problems.

#### Stastical analysis

Data analysis was performed using GraphPad Prism for Windows XP, version 4.0 (GraphPad Software, San Diego, CA, USA). Quantitative variables are shown as mean ± SD or median (range). The Mann–Whitney U-test was performed to examine the possible relationship between age, birth height and weight, height, weight, BMI, maternal, paternal and mid-parental heights and the parental origin of the X chromosome. The Kolmogorov-Smirnov test was used to test the normal distribution of these variables and Pearson correlation coefficients were used to assess the correlations among the various variables. To compare the frequencies of categorical variables between the Xp and Xm groups we performed the Fisher’s exact test. The Wilcoxon signed ranks test was used to relate the pre- and the on rhGH treatment patient variables. To estimate the parental origin effect on the response to rhGH treatment, multiple linear regression analysis was performed with growth response to rhGh treatment parameters as the dependent variables and parental origin of the retained X-chromosome as potentially influencing variable. The *t*-test was used to compare biochemical data between Xp and Xm patients. ANCOVA was used to adjust for age and BMI, as covariates followed by the Fisher protected least-significant-difference tests. Two-sided *p* values were calculated for age, BMI, total cholesterol, HDL-cholesterol, LDL-cholesterol, triglycerides and fasting glucose. Statistical significant differences were assumed if *p* < 0.05.

## Results

In Table 1 we can see the patient and parental data according to origin of the retained X-chromosome. No significant difference between the origin of the retained X-chromosome and height-SDS of patients was found (*p* < 0.31). A stronger, but non significant correlation (p ≤ 0.07) between final height of subjects carrying the Xm chromosome and maternal height was found (Table 2). There was also a trend towards significance between maternal height and patients height in the ≤ 7 years of age group (p ≤ 0.09) who retained the Xm. However, among the group of subjects who retained the Xp no correlation was demonstrated at any age group. The correlation observed between the patients’ height and mid-parental height was similar to the correlation seen between patients’ height and maternal height, but the statistical significance was less at each age group. We found no correlation at any age group between paternal and patients height in the Xp group.

**Table 1:** Patient and parental data according to origin of the retained X-chromosome

	***Xm (n = 67)***	***Xp (n = 26)***	***p***
Patients’ height-SDS	−3.8 ±1.32	−3.7 ±1.5	0.31
Maternal height SDS	*−1.1 ±0.9*	−0.8 ±0.7	0.14
Paternal height SDS	0.8 ±0.6	0.9 ±0.8	0.75
Mid-parental height	−0.9 ±0.9	−0.9 ±0.6	0.23

Values are expressed as group means ± SD, sample sizes as n =  .

**Table 2:** Correlation of patient’s height-SDS with parental height according to the retained X-chromosome at different age groups

**Age groups**	**Maternal height**		**Paternal height**		**Mid-parental height**	
	**Xm = 67**	**Xp = 26**	**Xm = 67**	**Xp = 26**	**Xm = 67**	**Xp = 26**
Neonatal	r = 0.341	r = 0.282	r = 0.301	r = 0.282	r = 0.263	r = 0.287
	p = 0.24	p = 0.45	p = 0.62	p = 0.39	p = 0.44	p = 0.43
≤ 7 years old	r = 0.58	r = 0.405	r = 0.381	r = 0.241	r = 0.489	r = 0.375
	p = 0.09	p = 0.11	p = 0.23	p = 0.43	p = 0.12	p = 0.21
Final Height	r = 0.601	r = 0.598	r = 0.362	r = 0.213	r = 0.521	r = 0.421
	p = 0.07	p = 0.09	p = 0.32	p = 0.52	p = 0.09	p = 0.17

Sample sizes as Xm =  or Xp=; and number of measures (in brackets). Kolmogorov-Smirnov test showed no deviation from linearity (p < 0.01). Values are correlations of patient and parental height (Pearson’s correlation coefficient) p ≤ 0.05.

No significant differences in the incidence of any of the visible somatic dysmorphic stigmata, or in urinary, cardiovascular, metabolic and skeletal abnormalities were observed among the patients from the different countries. Additionally, no significant differences between the subjects who retained the Xm or Xp chromosome were found in relation to the frequency of somatic stigmata or associated abnormalities (Table 3). The incidence of cardiovascular anomalies was similar between the two different genotype groups (18/67: 26.9% vs 8/26: 30.8%). Renal anomalies were present in 16/67 (23.9%) of the patients who retained the Xm versus 6/26 (23.1%) in those with retained Xp. There was no statistically significant difference among the genotypes described above (*p* =  0.9). Similar results were found for other anomalies, including ear and thyroid abnormalities (Table 3).

**Table 3:** Associated disorders and anomalies according to the parental origin of the retained X chromosome

**Type**	**Cardiac anomalies**		**Renal anomalies**		**Webbed neck**		**Ear disorders**		**Thyroid disorders**	
Parental origin	Xm	Xp	Xm	Xp	Xm	Xp	Xm	Xp	Xm	Xp
A/T: (%);	18/67^a^ (26.9)	8/26^a^ (30.8)	16/67^b^ (23.9)	6/26^b^ (23.1)	23/67^b^ (34.3)	9/26^b^ (34.6)	31/67^c^ (46.2)	12/26^c^ (46.1)	14/67^d^ (21)	6/26^d^ (23.1)

A/T: affected/total (%); Fisher’s exact test: ^a^p =  0.7980; ^b^p =  1.0, ^c^p =  0.1547; ^d^p =  0.7862.

Thirty-four of our TS patients received rhGH therapy, 24 of them retained the Xm, and 10 retained the Xp. At the beginning of therapy, there were no statistically significant differences in baseline auxological features between the two genotype groups. The chronological age was similar in both groups (Xm =  10.8 ± 2.8 years vs Xp =  9.9 ± 3.1 years ). The height-SDS of patients was not significantly different between those who retained the Xm (−2.81 ± 0.71) and those who retained the Xp (−2.66 ± 0.63). Growth hormone therapy significantly (p < 0.0001) increased growth velocity (cm/yr) and standing height (SDS and cm) during the first (7.56 ± 1.35 cm/yr; -2.47 ± 0.65) and second years (5.66 ± 1.51 cm/yr; -2.35 ± 0.71) in the total group (Xm + Xp). However, these changes were not significantly different among the patients who retained the Xm and those who retained the Xp (*p* value =0.51; 0.49; 0.26; 0.54; respectively) (Table 4). Additionally, no differences in the total two-year height gain or in the increment of height-SDS among patients with different retained X chromosome were found (Table 4).

**Table 4:** Clinical and auxological data of the growth response to rhGH according to the parental origin of the retained X chromosome

	**Xm (n = 24)**	**Xp (n = 10)**	**P**
Age at start yr	10.8 ± 2.8	9.9 ± 3.1	0.45
rhGH dose (mg/kg/day)	0,37 ±0.08	0,37 ± 0.02	0.87
Pretreatment height-SDS	−2.81 ± 0.71	−2.66 ± 0.63	0.12
HV during first yr (cm/y)	7.65 ± 1.73	7.87 ± 1.1	0.51
HV during second yr (cm/y)	5.74 ± 1.57	5.59 ± 1.77	0.49
Height-SDS at first yr	−2.21 ± 0.64	−2.25 ± 0.79	0.26
Δ height-SDS	0.55 ± 0.43	0.56 ± 0.41	0.54

According to SDS in relation to national growth chart references for each country. Multiple linear regression analysis was performed with growth response to rhGH treatment parameters as the dependent variables and parental origin of the retained X-chromosome as potentially influencing variable.

Pooled data from fasting serum levels of glucose, total-, LDL-, VLDL- and HDL-cholesterol were indistinguishable between the two genotype groups. Triglycerides, total-, HDL- and LDL-cholesterol and fasting glucose levels were similar in the Xm and Xp groups in subjects <20 years . However, serum levels of triglycerides (*p* < 0.001), total-(*p* < 0.03), and LDL-cholesterol (*p* < 0.04) were significantly higher in the subjects ≥20 years who retained the Xm chromosome, when compared to those in the the Xp group (Table 5).

**Table 5:** Serum lipid and carbohydrate data according to the parental origin of the retained X chromosome

	**< 20 years (n = 67)**			**≥ 20 years (n = 48)**		
	**Xm (n = 48)**	**Xp (n = 19)**	**P**	**Xm (n = 32)**	**Xp (n = 16)**	**p**
Total cholesterol (mmol/l)	175 ± 29	183 ± 35	0.21	199 ± 38	181 ± 41	0.03
HDL-cholesterol (mmol/l)	61 ± 15	62 ± 13	0.76	55 ± 11	61 ± 15	0.13
LDL-cholesterol (mmol/l)	98 ± 21	101 ± 15	0.65	105 ± 17	95 ± 21	0.04
Triglycerides (mmol/l)	95 ± 52	97 ± 45	0.61	127 ± 55	99 ± 52	0.001
Fasting glucose (mg/dl)	81 ± 9	85 ± 9	0.18	91 ± 8	88 ± 10	0.38
BMI	19.9 ± 1.34	20.7 ± 2.13	0.54	25.1 ± 7.3	24.7 ± 6.1	0.81

The means of Xm and Xp were compared by one-way ANCOVA analysis to age and BMI used as covariates, followed by Tukey’s significant-difference test. Two-sided *p* values were calculated for age, BMI, total cholesterol, HDL-cholesterol, LDL-cholesterol, triglycerides and fasting glucose, but were found to be significantly different only for total cholesterol (*p ≤* 0.001), and for LDL cholesterol (*p ≤* 0.037) in the ≥ 20-year-group.

## Discussion

As reported in previous studies [7-14], but with an appropriate sample size and with more sensitive/specific molecular analysis, we found that 2 out of 3 patients with monosomy for 45,X retained the Xm. To explain the higher frequency of maternal X-chromosome several hypotheses have been proposed [15-19]. However, and we agree with the hypothesis, given the nonviability of 45,Y cells, the reason for this ratio is that women have two chances of contributing to a monosomic offspring, while man has only one. Thus, the 2:1 ratio is not consistent with a protective effect of the maternal X-chromosome [8,9,20-22].

Altered lipid profiles have been reported in TS [23-26]. Apparently, this altered metabolic status was not associated to the karyotype or to BMI. Sagi et al. demonstrated that in their study group almost a third of the patients exhibited high total- and LDL-cholesterol levels. These atherogenic levels were significantly higher in the Xp than in Xm group [9]. Previously, Van et al. in adult TS patients and in a larger size study group demonstrated higher triglycerides, total and LDL-cholesterol in the Xm group than in the Xp groups [12]. Our study included the measurement of lipid profiles in both pediatric and adult patients, trying to clarify the discrepancy found by the two studies mentioned above. Our finding of an atherogenic lipid profile in older patients, significantly associated with the effect of the Xm, seems to support the hypothesis of a significant difference between the Xm and Xp groups which parallels the usual metabolic differences seen in normal women (46,XmXp) and men (46,XmY) [12]. Our findings suggests that the atherogenic lipid profile is intrinsic and gradually progressive in TS, as the majority of our adult TS patients were evaluated in different stages during both their pediatric and adult life. Therefore, the early onset of this atherogenic lipid profile may be influenced by the retained Xm chromosome. Another explanation for these findings is that lipid profiles are a highly heritable trait and the difference in lipid levels could be due to heritability. Performing lipid studies on the parents of the girls with TS would help clarify whether the difference in lipid levels is an effect of the maternal X rather than a correlation with parental lipid profiles.

Previous studies have shown conflicting results regarding the influence of the parental origin of the X-chromosome on the height of TS patients [7,9,11,13-15,20-22,27,28]. These differences may be due to the fact that most of the previous reports did not classify the patients according to their karyotypes and that patients SDS were calculated employing the growth reference charts of Lyon et al. [29], which could overestimate the patients’ height. Our study only analyzed patients with a 45,X karyotype and we used the national growth curves of each country as a reference to calculate the patients SDS. However, the differences noted between studies may be due mainly to confounding factors leading to common type I (false-positive results) errors which may be caused by a small sample size or due to insufficient power to generate reliable and reproducible results. The number of patients in the present study was quite large when compared with that of previous reports.

Several studies have suggested a positive correlation between the final height of TS patients and the height of their mothers and a significant correlation between maternal height and target height in Xm patients only [9,13,27,28]. However, studies by Hamelin et al. and Bondy et al. found no difference in height between the Xm and Xp groups, either during childhood or adulthood [11,14]. The best correlation we observed was between final height of subjects carrying the Xm chromosome and maternal height but this weak correlation was found to vary at different age group. Therefore, we interpret the data of the current study on the actual statistical results as 1) there is no important effect of the parental origin of the X-chromosome on height, and 2) maternal height influences the final height of TS patients regardless of the parental origin of the retained X-chromosome. In reality, the auxological data presented in the current study allows us to conclude that the effect of maternal X-chromosome imprinting on growth at different age groups in TS remains unclear and if any effect exists at all it is very minimal. A possible explanation for this minimal effect is that it plays a modifying role on (pseudo)autosomal genes that may be unmasked by aneuploidy 45,X or that the measurements from the same patient are no longer independent.

A Canadian study suggested that the parental origin of the retained X-chromosome is a factor involved in the response to rhGH in TS, as it explained more than 50% of the adult height gain achieved with rhGH therapy in Xm subjects [11]. In that study, the growth response to rhGH was slightly better in the Xm individuals (n =  26) probably because this group had mosaic karyotypes *(≈25%)* with high levels of normal cells, while the Xp group was small *(n =9)* and was entirely 45,X. On the other hand, Sagi et al. and Ko et al. found no evident differences in the response to rhGH between Xm and Xp groups [9,21]. Similar to these latter reports, we did not find significant differences between the Xm and Xp groups with respect to their response to rhGH. Nevertheless, our study has two limitations. First, the current study focused on the short-term response to rhGH (first and second years of rhGH therapy). The factors that influence the response in the first year of treatment may differ totally from those that lead to the prediction of the individuals’ final height. Secondly, only ten patients formed our Xp group and therefore our study may suffer from a low power to detect mild effects that may truly exist. Small size, in particular for the Xp group, is the major limiting factor in the comparison of the rhGH response according to the origin of the retained X chromosome in all published studies [9,11,21]. However, our findings in relation to the growth response to rhGH in TS are a product of a study that included a well defined group of 34 patients with a pure 45,X karyotype, conducted in six South American academic medical centers thus avoiding a possible selection bias or influence of local genetic pools; and the laboratory method is widely accepted. In addition, in our study the clinical characterization of the response to rhGh was prospective. Furthermore, a small sample size tends to give more impressive estimates, with false-positive associations, and unlike the Canadian study, no significant associations (negative or positive) were found between the phenotype variation and genotype in the current study. Recently, in the largest study carried out to date (n =  128 (Xm) vs n =  52 (Xp), Deverny et al. demonstrated the parental origin of the X-chromosome did not alter the effect of rhGH treatment [15]. Due to all these results, we propose that the parental origin of the retained X chromosome should not be taken into account when therapy with rhGH in TS patients is individualized.

Several studies have reported a higher incidence of cardiac or renal anomalies, webbed neck, ocular abnormalities and sensorineural hearing loss according to the retained X-chromosome [9,11-14,20-22,27]. We were, however, unable to explain the frequency or wide and variable difference observed in the phenotype of TS based on the retained X-chromosome. These contradictory findings may be due to the study design, small patient size, different and less sensitive methods utilized to detect anomalies, and/or the inclusion of different karyotypes in previous studies. Although cryptic mosaicism is preferably measured in peripheral blood lymphocytes, the possibility of cryptic mosaicism in tissues other than peripheral blood lymphocytes which could theoretically have altered the results, needs to be considered. The overall frequency of abnormalities found in our study (without taking into account the parental origin of the X-chromosome) was similar to the incidence reported in the literature. We agree with the hypothesis that it seems unlikely on biological grounds that X-chromosome-linked imprinted genes would be critically involved in the embryonic development of lymphatic, cardiovascular or renal systems, since these are not sexually dimorphic [14,30].

## Conclusions

The present study demonstrates that the parental origin of the X chromosome is unlikely to produce imprinting effects on visible somatic dysmorphic stigmata, urinary, cardiovascular and skeletal abnormalities and on the growth response to rhGH. The effect of maternal X-chromosome imprinting on growth in different age group in TS remains unclear and if any effect exists it is very minimal, suggesting a modifying role on growth (pseudo)autosomal genes. However, the parental origin-effect may be responsible for the expression of the X-chromosome-linked imprinted genes critically involved in lipid metabolism. The parental origin of the X chromosome must therefore be considered as a possible explanation for the significant differences in the lipid profile between Xm and Xp groups in TS patients with a 45,X karyotype which parallels differences usually noted between normal women and men.

## Abbreviations

X-STRs: X-chromosome single tandem repeats; rhGH: recombinant human growth hormone; TS: Turner syndrome; SDS: Standard deviation scores; HRT: Hormone replacement therapy; BMI: Body mass index; ANCOVA: Analysis of CoVariance; HDL-cholesterol: High density lipoprotein cholesterol; LDL-cholesterol: Low density lipoprotein cholesterol; VLDL-cholesterol: Very low density lipoprotein cholesterol; Xm: maternal X chromosome; Xp: paternal X chromosome.

## Competing interest

The authors declare that they have nothing to disclose and there is no conflict of interest that could be perceived as prejudicing the impartiality of the research reported.

## Authors’ contribution

The authors’ contribution to the paper is as follow: FAN and RL: study concepts and design, data analysis and interpretation, statistical analysis, obtaining funding, critical revision of the manuscript for important intellectual content and manuscript preparation; MS: study concepts and design, data analysis and manuscript preparation; JMQ, WZ, TP, LB, NU: molecular studies and data analysis; MM, HF, VM, JV, PG: clinical studies, acquisition of data and data analysis; and HM, NT, AB, LS, LF, ML, GF, MA, MS: clinical studies and acquisition of data. All authors read and approved the final manuscript.

## Supplementary Material

Additional file 1 Molecular Analysis. Click here for file
