# Supplementary material for: Effect of the parental origin of the X-chromosome on the clinical features, associated complications, the two-year-response to growth hormone (rhGH) and the biochemical profile in patients with turner syndrome
Source: Int J Pediatr Endocrinol. 2013 Jun 4;2013(1):10. doi: 10.1186/1687-9856-2013-10 (PMC3679778; doi:10.1186/1687-9856-2013-10)
Supplement: Additional file 1 — Molecular Analysis. [file 1687-9856-2013-10-S1.doc]

**Molecular Analysis**

**Isolation, Quantification and Purification Genomic DNA**

A few drops of venous blood from patients and their mothers were immediately spotted onto the preprinted circle of a filter paper. The filter paper was air-dried at room temperature for 4 h before storage in a paper bag. The entire dried blood spot was punched out in a 6mm in diameter section and a 3mm blood-spot was cut out using a sterile razor blade. All the samples were processed within the first 3 months of storage. Genomic DNA was extracted and quantitated using the DNA IQ™ System kit (Promega, Madison, WI, USA). All reagents were supplied by the manufacturer in the DNA IQ kit except for 95-100% ethanol and isopropyl alcohol.

**Selection of X-STRs**

PCR conditions were optimized in our laboratory for ten highly polymorphic X chromosome short tandem repeat (X-STRs) as previously described (1). Amplification was performed in a PCR multiplex reaction. These X-STRs were selected due to 1) their location on both Xp and Xq: 2 markers were distributed along the short arm of X (DXS8378; Xp22.31, DXS9902;Xp22.2), and the other 8 markers were located in the long arm of the X chromosome (DXS9898;Xq21.31, DXS7131;Xq21.1, GATA31E08; Xq27.1, GATA172D05; Xq23, DXS7423;Xq28, DXS6809; Xq21.33, DXS7132;Xq11.2 and DXS6789;Xq21.33); 2) their high degree of heterozygosity (between 66 to 82%); and 3) their high discrimination power obtained in Latin-American populations, both in males (≥1 in 5×10^5^) and females (≥1 in 3×10^9^), as well as high mean exclusion chance in father/daughter duos (≥99.953%) and in father/mother/daughter trios (≥99.999%) (2).

**PCR Amplification**

PCR conditions were optimized in our laboratory using the QIAGEN Multiplex PCR kit according Gusmao et al at 2×Qiagen multiplex PCR master mix and 0.5–5 ng of genomic DNA in a 10-μl final reaction volume (1). All primers were at 0.2 μM in the PCR reaction. Thermocycling conditions were: pre-incubation for 15 min at 95°C, followed by ten cycles of 30 s at 94°C, 90 s at 60°C, 60 s at 72°C; and 20 cycles of 30 s at 94°C, 90 s at 58°C, and 60 s at 72°C with a final incubation for 60 min at 72°C in a in a thermal cycler MJ-Research model.

**Fragments analysis**

Separation and detection of the different amplicons generated during the PCR was performed in an ABI PRISM 310 Genetic Analyzer (Applied Biosystems) following manufacturer instructions. The decaplex X-STRs was optimized for typing with the internal size standard LIZ500 (Applied Biosystems). The allelic determination was developed using the genemapper software (Applied Biosystems) and for quality control a DNA sample of reference was used as an internal control in each case, so as to corroborate the relative sizes of amplified fragments. The parental origin of the normal X chromosome was determined by genotype comparisons between patients with TS and their mothers.

**REFERENCES**

1. [**Gusmão L**](http://www.ncbi.nlm.nih.gov/pubmed?term="Gusmão L"%5BAuthor%5D)**,** [**Sánchez-Diz P**](http://www.ncbi.nlm.nih.gov/pubmed?term="Sánchez-Diz P"%5BAuthor%5D)**,** [**Alves C**](http://www.ncbi.nlm.nih.gov/pubmed?term="Alves C"%5BAuthor%5D)**,** [**Gomes I**](http://www.ncbi.nlm.nih.gov/pubmed?term="Gomes I"%5BAuthor%5D)**,** [**Zarrabeitia MT**](http://www.ncbi.nlm.nih.gov/pubmed?term="Zarrabeitia MT"%5BAuthor%5D)**,** [**Abovich M**](http://www.ncbi.nlm.nih.gov/pubmed?term="Abovich M"%5BAuthor%5D)**,** [**Atmetlla I**](http://www.ncbi.nlm.nih.gov/pubmed?term="Atmetlla I"%5BAuthor%5D)**,** [**Bobillo C**](http://www.ncbi.nlm.nih.gov/pubmed?term="Bobillo C"%5BAuthor%5D)**,** [**Bravo L**](http://www.ncbi.nlm.nih.gov/pubmed?term="Bravo L"%5BAuthor%5D)**,** [**Builes J**](http://www.ncbi.nlm.nih.gov/pubmed?term="Builes J"%5BAuthor%5D)**,** [**Cainé L**](http://www.ncbi.nlm.nih.gov/pubmed?term="Cainé L"%5BAuthor%5D)**,** [**Calvo R**](http://www.ncbi.nlm.nih.gov/pubmed?term="Calvo R"%5BAuthor%5D)**,** [**Carvalho E**](http://www.ncbi.nlm.nih.gov/pubmed?term="Carvalho E"%5BAuthor%5D)**,** [**Carvalho M**](http://www.ncbi.nlm.nih.gov/pubmed?term="Carvalho M"%5BAuthor%5D)**,** [**Cicarelli R**](http://www.ncbi.nlm.nih.gov/pubmed?term="Cicarelli R"%5BAuthor%5D)**,** [**Catelli L**](http://www.ncbi.nlm.nih.gov/pubmed?term="Catelli L"%5BAuthor%5D)**,** [**Corach D**](http://www.ncbi.nlm.nih.gov/pubmed?term="Corach D"%5BAuthor%5D)**,** [**Espinoza M**](http://www.ncbi.nlm.nih.gov/pubmed?term="Espinoza M"%5BAuthor%5D)**,** [**García O**](http://www.ncbi.nlm.nih.gov/pubmed?term="García O"%5BAuthor%5D)**,** [**Malaghini M**](http://www.ncbi.nlm.nih.gov/pubmed?term="Malaghini M"%5BAuthor%5D)**,** [**Martins J**](http://www.ncbi.nlm.nih.gov/pubmed?term="Martins J"%5BAuthor%5D)**,** [**Pinheiro F**](http://www.ncbi.nlm.nih.gov/pubmed?term="Pinheiro F"%5BAuthor%5D)**,** [**João Porto M**](http://www.ncbi.nlm.nih.gov/pubmed?term="João Porto M"%5BAuthor%5D)**,** [**Raimondi E**](http://www.ncbi.nlm.nih.gov/pubmed?term="Raimondi E"%5BAuthor%5D)**,** [**Riancho JA**](http://www.ncbi.nlm.nih.gov/pubmed?term="Riancho JA"%5BAuthor%5D)**,** [**Rodríguez A**](http://www.ncbi.nlm.nih.gov/pubmed?term="Rodríguez A"%5BAuthor%5D)**,** [**Rodríguez A**](http://www.ncbi.nlm.nih.gov/pubmed?term="Rodríguez A"%5BAuthor%5D)**,** [**Rodríguez Cardozo B**](http://www.ncbi.nlm.nih.gov/pubmed?term="Rodríguez Cardozo B"%5BAuthor%5D)**,** [**Schneider V**](http://www.ncbi.nlm.nih.gov/pubmed?term="Schneider V"%5BAuthor%5D)**,** [**Silva S**](http://www.ncbi.nlm.nih.gov/pubmed?term="Silva S"%5BAuthor%5D)**,** [**Tavares C**](http://www.ncbi.nlm.nih.gov/pubmed?term="Tavares C"%5BAuthor%5D)**,** [**Toscanini U**](http://www.ncbi.nlm.nih.gov/pubmed?term="Toscanini U"%5BAuthor%5D)**,** [**Vullo C**](http://www.ncbi.nlm.nih.gov/pubmed?term="Vullo C"%5BAuthor%5D)**,** [**Whittle M**](http://www.ncbi.nlm.nih.gov/pubmed?term="Whittle M"%5BAuthor%5D)**,** [**Yurrebaso I**](http://www.ncbi.nlm.nih.gov/pubmed?term="Yurrebaso I"%5BAuthor%5D)**,** [**Carracedo A**](http://www.ncbi.nlm.nih.gov/pubmed?term="Carracedo A"%5BAuthor%5D)**,** [**Amorim A**](http://www.ncbi.nlm.nih.gov/pubmed?term="Amorim A"%5BAuthor%5D) 2009 A GEP-ISFG collaborative study on the optimization of an X-STR decaplex: data on 15 Iberian and Latin American populations. [Int J Legal Med](http://www.ncbi.nlm.nih.gov/pubmed?term=Gusmão L%2C Sánchez-Diz P%2C Alves C%2C Gomes I%2C Zarrabeitia MT%2C Abovich M%2C Atmetlla I%2C Bobillo C%2C Bravo L%2C Builes J%2C Cainé L%2C Calvo R%2C ) 123:227-234.
2. [**Edelmann J**](http://www.ncbi.nlm.nih.gov/pubmed?term="Edelmann J"%5BAuthor%5D)**,**[**Deichsel D**](http://www.ncbi.nlm.nih.gov/pubmed?term="Deichsel D"%5BAuthor%5D)**,**[**Hering S**](http://www.ncbi.nlm.nih.gov/pubmed?term="Hering S"%5BAuthor%5D)**,**[**Plate I**](http://www.ncbi.nlm.nih.gov/pubmed?term="Plate I"%5BAuthor%5D)**,**[**Szibor R**](http://www.ncbi.nlm.nih.gov/pubmed?term="Szibor R"%5BAuthor%5D) 2002 Sequence variation and allele nomenclature for the X-linked STRs DXS9895, DXS8378, DXS7132, DXS6800, DXS7133, GATA172D05, DXS7423 and DXS8377. [Forensic Sci Int](http://www.ncbi.nlm.nih.gov/pubmed?term=Edelmann J%2C Deichsel D%2C Hering S%2C Plate I%2C Szibor R.) 129:99-103.
